# Air Quality in Alternative Housing Systems May Have an Impact on Laying Hen Welfare. Part I—Dust

**DOI:** 10.3390/ani5030368

**Published:** 2015-07-09

**Authors:** Bruce David, Randi Oppermann Moe, Virginie Michel, Vonne Lund, Cecilie Mejdell

**Affiliations:** 1 Norwegian Veterinary Institute, P.O. Box 750 Sentrum, Oslo 0106, Norway; E-Mail: cecilie.mejdell@vetinst.no; 2 Norwegian University of Life Sciences, P.O. Box 8146 Dep., Oslo 0033, Norway; E-Mail: randi.moe@nmbu.no; 3 French Agency for Food, Environmental and Occupational Health Safety (Anses), P.O.Box 53, Ploufragan 22440, France; E-Mail: virginie.MICHEL@anses.fr

**Keywords:** furnished cages, loose housing, aviaries, behavior, health, laying hens

## Abstract

The new legislation for laying hens in the European Union put a ban on conventional cages. Production systems must now provide the hens with access to a nest, a perch, and material for dust bathing. These requirements will improve the behavioral aspects of animal welfare. However, when hens are kept with access to litter, it is a concern that polluted air may become an increased threat to health and therefore also a welfare problem. This article reviews the literature regarding the health and welfare effects birds experience when exposed to barn dust. Dust is composed of inorganic and organic compounds, from the birds themselves as well as from feed, litter, and building materials. Dust may be a vector for microorganisms and toxins. In general, studies indicate that housing systems where laying hens have access to litter as aviaries and floor systems consistently have higher concentrations of suspended dust than caged hens with little (furnished cages) or no access to litter (conventional cages). The higher dust levels in aviaries and floor housing are also caused by increased bird activity in the non-cage systems. There are gaps in both the basic and applied knowledge of how birds react to dust and aerosol contaminants, *i.e.*, what levels they find aversive and/or impair health. Nevertheless, high dust levels may compromise the health and welfare of both birds and their caretakers and the poor air quality often found in new poultry housing systems needs to be addressed. It is necessary to develop prophylactic measures and to refine the production systems in order to achieve the full welfare benefits of the cage ban.

## 1. Introduction

The welfare of laying hens is of great concern to the European Commission, citizens, and the egg industry [1,2]. The council directive 1999/74/EC put a ban on conventional cages in EU from 2012. The directive improves laying hen welfare, especially their freedom to express behavioral priorities such as laying eggs in a nest, dust bathing, and perching in enriched cages; and, in addition, wing flapping and mobility in loose housing systems. There is no doubt that such allowances have a positive effect on vital aspects of animal welfare in laying hens. These welfare benefits are well documented in several scientific studies summarized by EFSA [1]. Canada and the United States are also considering a transition away from battery cages [3,4].

However, the approved systems currently available for intensive large-scale egg production may have negative side effects that cause other welfare problems, like increased risk of feather-pecking and cannibalism, especially within non-cage systems [1]. Another important challenge and the theme of two review articles in the current special issue on poultry welfare is the aerial environment.

This first article reviews the effects of air quality on the welfare of laying hens with regards to airborne dust. In order to discuss the effects of aerial pollutants on the welfare of poultry, an introduction to the concept of animal welfare is given. The aim is to ease the understanding of why different aspects of welfare are valued differently by different people. Thereafter a brief description of specific features of the avian respiratory system is given to better understand the effects of air pollutants. The effects of ammonia in poultry houses are discussed in Part II: “Air Quality in Alternative Housing Systems May Have an Impact on Laying Hen Welfare. Part II—ammonia” in this journal [5]. Where little research has been published regarding laying hens, we refer to research done on other types of poultry, mainly broilers.

## 2. The Concept of Animal Welfare

The Brambell Committee was one of the first to suggest a definition of animal welfare. This definition later became known as “the five freedoms”: freedom (1) from hunger and thirst; (2) from discomfort; (3) from pain, injury, or diseases; (4) to express normal behaviors; and (5) from fear and distress [6,7]. The World Organization for Animal Health (OIE) has adopted the following definition: “Animal welfare means how an animal is coping with the conditions in which it lives. An animal is in a good state of welfare if (as indicated by scientific evidence) it is healthy, comfortable, well nourished, safe, able to express innate behavior, and is not suffering from unpleasant states such as pain, fear, and distress. Good animal welfare requires disease prevention and veterinary treatment, appropriate shelter, management, nutrition, humane handling, and humane slaughter/killing” [8]. According to Dawkins [9], assessment of animal welfare should be directed at answering two questions: Are the animals healthy? Do they get what they want?

Depending on how animal welfare is understood, the underlying values of the interpreters, the selection of indicators to study, and how differing and even contradicting evidence is weighed may result in different conclusions regarding animal welfare [10,11].

Regarding laying hens, a main challenge is that increasing environmental complexity to improve the hens’ possibility to express motivated behaviors could result in health problems and, consequently, reduce welfare. In this special edition on poultry welfare, we describe the effects of dust (reported here) and ammonia [5] on laying hen welfare, and we have focused on health implications and behavioral studies related to the aerial environment in furnished cages and different loose housing systems.

## 3. Anatomy of the Avian Respiratory System

The structure of the avian respiratory system is unique among vertebrates [12]. Research on the effects of air quality in mammals can therefore not be directly applied to poultry. Birds have small lungs that do not change volume during breathing and nine large air sacs that act as bellows to ventilate the lungs, while not directly participating in gas exchange [12]. The functional anatomy of the avian respiratory system is very effective, allowing for gas exchange in the lungs both during inhalation and exhalation. Birds can breathe through either the nares or the mouth. The trachea has complete cartilaginous rings, in contrast to the incomplete rings of mammals, which are connected with a collapsible membrane. This is considered an important feature of the mammalian cough, an effective mechanism for clearing debris from the upper airway. It is uncertain whether the complete rings of the bird reduce the effectiveness of coughing for clearing the passageways, but birds are observed to cough [13]. Each of the primary bronchi enters the lung, and exits caudally into an abdominal air sac. Each primary bronchus gives rise to four groups of secondary bronchi. Numerous tertiary bronchi (parabronchi) branch off within the lung [14]. Gaseous exchange occurs in tiny air capillaries that form extensive networks interconnecting the parabronchi, and there are no dead-end structures comparable to the alveoli of mammals [13]. The transparent walls of the air sac are composed of a network of elastic fibers with some collagen. The air sacs have systemic arterial supply but are poorly vascularized [13,15].

## 4. Categories, Sources, and Composition of Dust

Exposure to dust is commonly found to be greater in poultry houses compared to other animal productions [16,17,18,19].

Dust is composed of small particles. The finer particles may easily be suspended in air. The size of dust particles is often defined in terms of inhalable, thoracic, and respirable dust [20,21]. This description is based upon its ability to reach the different parts of mammalian lungs. Using these definitions, a respirable particle has a diameter of less than 4 [21] or 5 μm [20] and may pass all the way down to the alveoli. Thoracic particles (5–10 μm) may pass the larynx and be found in the bronchioles. Inhalability decreases gradually with increasing particle diameter. Inhalable particles are usually considered to be bigger particles with diameters up to 100 μm. Inhalable particles may accumulate in the nostrils and in the nasal cavity. In intensive animal production housing, the respirable fraction typically represents 5% to 10% of the inhalable particles [20].

It is debatable to what extent these definitions are valid for poultry. Early investigations of the anatomy and mechanisms of the avian respiratory system used soot to determine the unidirectional gas flow through the lungs [22,23]. The soot particles were deposited first in the caudal portion of the respiratory system, thus being respirable. In 1982, these findings were confirmed using particles with a diameter of 0.45 μm [24]. Later investigations in poultry have determined that dust particles with a diameter from 3.7 to 7 μm settle in the anterior portions of the respiratory tract and thus are inhalable [25]. Smaller particles (1.1 to 0.091 μm) may be found equally throughout the respiratory tract, including the lungs and the air sacs [25], and can be described as respirable for poultry. Based on this, respirable dust has a smaller diameter in birds compared to mammals. Corbanie *et al.* [26] performed experiments to determine the effect of age on how far particles of different sizes penetrated the respiratory system in broiler chicks. In 2- and 4-week-old chicks, particles with a size of 5 and 10 μm, respectively, were too large to reach the lungs and air sacs. The difference found here is probably due to the differing size of the airways. However, in day-old chicks particles up to 20 μm were found to reach the lungs and air sacs. A possible explanation for this apparent contradiction is that the young chicks breathe through their mouths initially and therefore do not benefit from the filter effect of the nares. This would also be a relevant situation in hot conditions where birds pant with their mouths open in order to dissipate heat.

Dust in the poultry house is of both organic and inorganic origin, with birds and their sheddings being the main source [27]. The inorganic dust originates from building materials such as concrete, metal, mineral or fiberglass insulation, or material such as soil particles brought into the house by the fresh air supply. In a study of a broiler facility, calcium from the feed was found to be the most common inorganic dust component [28]. Magnesium, copper, iron, lead, and zinc were other feed or cage components found in the airborne dust. However, in general, publications considering dust in animal housing have focused on organic dust.

Included in the organic dust of poultry houses are feather and skin particles, feed components, dried fecal matter, molds, fungi, bacteria and bacterial endotoxins, and viruses [29]. Koon *et al.* [30] found that the organic dust from caged layers consisted of two distinct types of particulate matter. The bulk of the matter was flat, flaky, and cellular in structure, with a diameter from 1 to 450 μm, some containing droplets of oil, and was identified as skin debris and feed particles. The other matter was long, cylindrical particles with nodes and internodes, identified as broken feather barbules. Both types of dust contained electrostatic charges, causing them to clump and form aggregates. The dust contained approximately 92% dry matter, of which 60% was crude protein, 9% fat, 4% cellulose, and the rest ash and hydrocarbons.

Airborne particulates, referred to as bioaerosols, including bacteria, endotoxins, viruses, and fungi, are also present in poultry houses [31,32], with dust often acting as a mechanical vector. Bacteria include non-pathogenic and dead bacteria. Gram-positive bacteria are more widely represented than Gram-negative in dust. Most authors have measured the total number of cultivable microorganisms and less often quantify and identify the genus and species [33]. The main microorganism genera identified are, for bacteria: *Bacillus*, *Clostridia*, *Corynebacterium*, *Enterobacter*, *Flavobacterium*, *Pseudomonas*, *Staphylococcus*, and *E. coli*; and for fungi: *Cladosporium*, *Penicillium*, and *Aspergillus* are the most common, but *Alternaria*, *Fusarium*, *Geotrichum*, and *Streptomyces* can also be present [20,34,35].

A number of the constitutive parts of microorganisms are found in dust. These include cell membrane peptidoglycans and other peptides (proteases, heat-shock proteins, *etc.*) as well as endotoxins from Gram-negative bacteria and mycotoxins from fungi [20,36,37]. An experimental study by Michel *et al.* [38] found low levels of mycotoxins such as tricothecene B, deoxynivalenol, and zearalenone in suspended dust. The likely source of the mycotoxins was feed, ingested and excreted in droppings.

## 5. Dust and Dust Components in Different Housing Systems

An overview of the literature regarding specific dust levels and dust components found in various housing systems for laying hens is summarized in Table 1.

The papers mostly report respirable dust rates ranging from 0.1 mg/m^3^ in conventional cages to a maximum of 1.19 mg/m^3^ in aviaries. Floor housing represents intermediate figures (0.37–0.848 mg/m^3^). Dust levels are apparently similar in furnished and conventional cages. This could be explained by litter not always being provided in furnished cages, as witnessed in field studies [39]. Reported levels of total and inhalable dust vary, even within the same system category, depending on the study. The level of total dust is found to be higher in floor systems compared to cages (e.g., around 12 mg/m^3^ in floor systems and 2.4 mg/m^3^ maximum in cages). Endotoxins are reported in very variable levels and comparison across studies is difficult due to the use of different units of measure (ng/m^3^ or EU/m^3^). Nevertheless, endotoxin levels, as dust levels, appear to be more important in alternative systems compared to cage systems. The same trend is seen for bacteria, with a specific increase in aviaries.

**Table 1:** Dust levels and components found in various systems for laying hens.

Reference			Dust Components		Measurement Technique				Conventional Cage			Furnished Cage		Floor Housing				Aviary/ Perchery	
Zhao *et al.* [40]			Inhalable dust (mg/m^3^)		TEOM				0.59			0.44		-				3.95	
			Respirable dust (mg/m^3^)		TEOM				0.035			0.056		-				0.41	
Le Bouquin *et al.* [39]			Respirable dust (mg/m^3^)		CIP 10				-			0.13		0.37				1.19	
Huneau-Salaün *et al.* [41] *			Endotoxins–experimental (EU/m^3^)						-			98 (51–470)		-				565 (362–1491)	
			Endotoxins- field measures (EU/m^3^)						-			78–576		-				35–3156	
Rimac *et al.* [42]			Total dust (mg/m^3^)		SKC pump				-			0.35		-				-	
			Total fungi (cfu/m^3^)						-			1.27 × 10^4^		-				-	
			Endotoxin (EU/m^3^)						-			233.8		-				-	
Nimmermark *et al.* [43]			Total dust (mg/m^3^)						-			2.3 (2.05–2.48)		12 (6.84–17.65)				1.8 (0.71–2.58)	
			Bacteria (10^7^ cells/m^3^)						-			1.6 (1.1–2.2)		8.8 (8.0–9.6)				2.8 (2.2–3.4)	
Saleh [44] *			Inhalable dust (mg/m^3^)		IOM				1.22 (0.24–2.27)			1.5 (0.44–3.48)		-				3.69 (1.3–9.5)	
			Respirable dust (mg/m^3^)		Cyclone				0.34 (0.01–1.3)			0.24 (0.01–0.99)		-				1.67 (0.2–4.4)	
			Bacteria (cfu/m^3^)		IOM				5.1 (0.2–22)			1.7 (0.09–4.1)		-				25 (5.1–81)	
			Fungi (cfu/m^3^)		IOM				1177 (90–7226)			1490 (140–20,395)		-				2455 (142–10,885)	
			Inhalable endotoxins (EU/m^3^)						373 (47–1222)			865 (50–3303)		-				1992 (237–3623)	
			Respirable endotoxins (EU/m^3^)						328 (9–759)			80 (5–243)		-				971 (18–1827)	
Michel *et al.* [38] *			Dust (mg/m^3^)		CIP 10				1			-		-				5–14	
			Trichothecene (μg/kg)						50			-		-				20–30	
			Deoxynivalenol (μg/kg)						60–320			-		-				20–80	
			Zearalanone (μg/kg)						-			-		-				45	
Saleh *et al.* [45]			Bacteria winter (cfu/m^3^ × 10^6^)		IOM				0.25			-		0.39				2.16	
			Bacteria summer (cfu/m^3^ × 10^6^)		IOM				0.38			-		0.12				0.56	
Michel and Huonnic [46] *			Bacteria (log cfu/m^3^)						1.35			-		-				3.8	
Ellen *et al.* [47]			Total dust (mg/m^3^)						1.51			-		7.33				7.6	
Seedorf *et al.* [17]			Endotoxins (ng/m^3^)-UK		IOM				549.2			-		2815.9				-	
			Endotoxins (ng/m^3^)-NL		IOM				20.8			-		431.3				-	
			Endotoxins (ng/m^3^)-DK		IOM				116.0			-		265.3				-	
Larsson *et al.* [48]			Total dust		IOM				2.4			-		-				4.1;4.8	
			Endotoxins						106			-		-				96;125	
Takai *et al.* [49]			Inhalable dust daytime (mg/m^3^)		IOM				1.51			-		-				7.33	
			Inhalable dust nighttime (mg/m^3^)		IOM				0.86			-		-				2.82	
Takai *et al.* [50]			Inhalable dust (mg/m^3^)		IOM				1.22 (0.75–1.64)			-		-				-	
			Respirable dust (mg/m^3^)		Cyclone				0.14 (0.03–0.23)			-		-				-	
Wathes *et al.* [51]			Inhalable dust (mg/m^3^)		IOM				1.7			-		-				2.8	
			Respirable dust (mg/m^3^)		Cyclone				0.1			-		-				0.17	

Dust measurement techniques; TEOM (Model 1400a, Thermo Fisher Scientific Inc., Waltham, MA USA), CIP 10 (Inhalable and respirable models, ARELCO, France), IOM (personal dust sampler, SKC Inc., Eighty Four, PA, USA), Cyclone (Respirable dust sampler, SKC Inc.); all measurements were taken in the animal room. * denotes references reporting results based on experimental facilities.

To sum up, dust levels and the bioactive components of dust are generally found to be much higher in loose housing systems compared to cages. Only two studies [40,44] have compared conventional and enriched cages, and they found no significant difference between these two cage systems. However, in one of the two studies [40], no litter was available for the hens in the cage.

## 6. Factors Affecting Dust Levels

The amount of airborne dust in a hen house depends upon the animals’ access to litter, its quality, and the birds’ activity level. These factors are greatly influenced by the housing system [17,46,47,52,53]. Furthermore, the concentration of suspended dust decreases in direct proportion to the height above the floor [52]. As a result, floor-raised birds are exposed to the highest concentrations of dust in the room, much higher than those experienced by the poultry workers.

Houses where broiler chickens are housed with access to litter have higher numbers of both respirable (<5 μm) and larger particles suspended in the air compared to keeping them on a netting floor [30,52]. Studies indicate that housing systems where laying hens have access to litter—especially floor housing systems and aviaries—consistently have higher concentrations of suspended dust and its components compared to cage systems where hens have little or no access to litter [38,39,40,41,43,45,46,47,53,54]. This is as expected, considering the fact that a large proportion of the dust in loose housing systems originates from the litter.

Peaks of dust concentrations are measured during times when the birds are very active, because their activity raises settled dust [55]. In aviary systems dust levels have been shown to be significantly higher in the afternoon than in the morning, owing to the hens’ dust-bathing behavior [47,56,57], and also after the light is turned on [41]. In general, laying hens are more physically active at higher light intensities [41,58]. Similarly, the feeding system and management may influence bird activity, and therefore affect the concentration of suspended dust. Schierl *et al.* [59] found that levels of suspended endotoxins had a diurnal variation, with daytime concentration being 14 times that of nighttime. The authors attributed this to animal activity when feeding. Poultry species, breed, and age affects activity. Consequently, suspended dust concentrations are usually low in broiler chicken flocks (relatively inactive birds), and higher in pullet loose-rearing (more active) and in guinea fowl (very nervous and active) [60]. The activity of the stockperson also plays a role in the suspension of dust [38,61].

Another important factor affecting dust levels is the relationship between temperature, humidity, and activity. Both in cages and in loose housing systems, average aerial dust concentrations have been found to be positively correlated with indoor air temperature and negatively to relative humidity [41]. Koon *et al.* [30] found that the quantity of dust produced by caged layers was low at 10 °C (50 °F), increased to a high level at 16 and 21 °C (60 and 70 °F), and then decreased as the temperature approached 38 °C (100 °F). The authors suggested that this was caused by increased bird activity at the medium temperature. For broilers kept on litter, there was, on the contrary, a distinct decline in dust production for birds kept at 32 °C (90 °F) compared to 16 and 24 °C (60 and 75 °F); the authors attributed this to an increase in absolute humidity (the relative humidity was the same at all temperatures) [30]. Also, according to Grub *et al.* [62], dust production by layers on litter is a function of e.g., air moisture. The finding that dust levels dropped as air moisture increased appears to support this conclusion with regards to floor systems, but this was not the case in barren cages [30]. An explanation for this discrepancy might be that dust in litter systems contains absorbent particles from wood shavings, which when moist will cause aggregation and settling.

Dust levels may also be affected by the use of ventilation to maintain a precise temperature inside the house [38]. The aerial concentration of bacteria decreases in the summer [45], probably because of the increased ventilation rate. Practical experience indicates that the dust-reducing effect of ventilation varies between buildings, and that commonly used air-mixing ventilation systems may not be able to reduce dust levels significantly (Nimmermark, pers. comm.).

Gustafsson and von Wachenfelt [63] reported that the type of litter material to some extent affected dust levels in loose housing systems; gravel resulted in higher dust levels compared to chopped straw, peat, and wood shavings. Dust bathing behaviors in furnished cages were more frequently seen in baths with sawdust than with sand [64]; however, no publication was found on the effects of various types of litter and litter management in furnished cages in relation to air quality.

## 7. Consequences for Birds

### 7.1. Health

Dust may have direct and indirect negative health effects and, thus, affect welfare [65].

Airborne microorganisms are frequently attached to dust particles. These microorganisms may be directly pathogenic or release toxins, meaning that dust in a poultry house may serve as a pathogen disseminator in addition to making the animals more susceptible to normally non- or low-pathogenic microorganisms. According to Wolfe *et al.* [66], dust increased the number of turkey condemnations at slaughter due to infections of the air sacs. Broilers raised on litter were also observed to have a higher incidence of lung damage ascribed to infection than that of broilers raised on netting floors [52]. Microorganisms following non-respirable dust clogging up the birds’ head may cause infections in the nares and upper respiratory tract [52].

Many of the organic dust particles are antigenic and can activate both the innate and the adaptive immune systems. This antigenicity may result in an inflammation of the exposed areas. Antigens and allergens that can induce allergic reactions include mites, pollen, fungi, and even components of animal origin in the farm environment [36,67,68].

Human and animal studies have demonstrated that exposure to organic dust can sensitize the lungs and may lead to hypersensitivity reactions [37,69] and respiratory diseases. Dust may impair lung clearance mechanisms and depress immune response to infection [69,70,71,72]. Michel and Huonnic [46] found pulmonary lesions of parabronchitis at the end of laying period to be more extensive and severe in birds in aviaries than in caged hens. This was thought to be a result of the differing dust concentrations, with respective maximum levels of 31.6 mg/m^3^ and 2.3 mg/m^3^. Riddell *et al.* [72], found that when comparing warm (27 °C) and cool (16 °C) poultry houses, more than 50% of the broiler chickens in warm rooms had microscopic lesions in the bronchi of their lungs, whereas fewer than 5% of chickens in cold rooms had such lesions. Large dust particles were visible in some of the lesions. The increased incidence of lung lesions in chickens from warm rooms was interpreted to be due to mouth-breathing rather than being a result of the higher dust levels in the air of these rooms. The mouth-breathing allowed the dust to penetrate deeper into the respiratory system by bypassing the natural filtration of the sinuses.

Dust might make the respiratory system more susceptible to even non-pathogenic microorganisms. Oyetunde *et al.* [73] showed that normally harmless *E. coli* had pathogenic effects on the respiratory system of four-week-old chicks when combined with sterile dust with a mean concentration of 101 mg/cm^3^ to 103.72 mg/cm^3^.

Interestingly, despite the fact that Madelin and Wathes [52] found a higher load of dust and microorganisms in litter houses, and also a higher incidence of lung damage and living microorganisms present in the broilers’ lungs at necropsy, there was no significant effect on mortality. Actually the birds raised on litter tended to have lower mortality. More air sac lesions and even lower mortality was found for turkeys kept on litter [74].

### 7.2. Behavior

No studies have been found describing the effects of dust on poultry behavior. However, it cannot be ruled out that birds, like humans, experience discomfort when dust clogs the upper respiratory passages and causes irritation of the eyes and nose.

### 7.3. Production

Whereas high production does not necessarily imply good welfare, reduced production may indicate a welfare problem [65]. Only one study has been found dealing with the effect of dust on production parameters. Madelin and Wathes [52] found significantly better food utilization in broiler chickens kept on litter compared to a netting floor, despite a higher load of dust and microorganisms in litter houses.

## 8. Discussion

The alternative housing systems for laying hens undoubtedly provide the animals with resources highly important to their welfare, as reviewed by EFSA [1]. For example, access to litter is of utmost importance for hens to display motivated behaviors such as dust bathing or scratching. At the same time, access to litter is also a classical example of the dilemma arising when solving a welfare concern, e.g., by allowing motivated behaviors, may give rise to other welfare problems, e.g., due to poor air quality. The challenge is to keep the welfare benefits of the alternative systems while avoiding other welfare problems. 

This paper offers a review of the available literature on potential animal welfare challenges related to dust in the housing systems for laying hens that became compulsory in 2012. There is considerable evidence that providing laying hens with litter material increases the amount of suspended dust in the poultry house. Not surprisingly, a high dust level in the new systems is also a concern for the health of poultry workers [41]. Large litter areas and high activity levels contribute to generally high levels of suspended dust in loose housing systems. There are very few studies that have looked at dust levels in furnished cages, but in general, dust levels seem to be lower in these than in loose housing systems and comparable to those in conventional cages. There are several possible explanations for this: the activity level of the birds is lower in cages than in loose housing systems, it is common (and legal) to restrict birds’ access to the dust bathing area to prevent misplaced eggs, and not all farmers renew the dust bathing material regularly, if at all. In addition, studies have revealed that dust bathing behavior in enriched cages is often disrupted [75] and a large proportion (30%) of the birds never enter this compartment [65]. In some litter box designs, the litter is displaced from the compartment during dust bathing, resulting in the litter not being available for the next bird. This may be one explanation for the lower dust levels in furnished cage systems. This has led to the question of whether the furnished cages as they are designed and managed do fulfill the individual bird’s dust bathing motivation. This illustrates the need for system refinement.

The literature review shows that dust levels are sometimes high, also the inhalable and respirable fractions. This is also the case for pollutants attached to dust particles such as bacteria, endotoxins, fungi, and mycotoxins. Sensitization or depression of the immune function and even lesions in the respiratory system, as a result of exposure to dust, have been demonstrated. The intensity of the experienced discomfort caused by dust is difficult to evaluate. Although the discomfort may not be very severe, the duration of exposure is relevant to animal welfare and should be considered, especially considering the longer life span of laying hens compared to meat poultry. Thus, health problems that have been documented in broiler chickens and turkeys may be an even greater welfare problem for laying hens.

The benefits and drawbacks of the system in question should not be considered inherent and unchangeable. Rather, the superior aspects of each system and the cause of the major problems in that system should be assessed and understood. In this respect, it is interesting to note that environmental enrichment (litter *versus* netting floor) led to reduced mortality and enhanced productivity despite the documented pathological changes in the respiratory system [53]. This illustrates the complexity of the interactions between animals and their environment.

The new legislation requiring access to a nest, litter, and perches has a profoundly positive effect on vital aspects of animal welfare in laying hens [1], in particular when emphasizing the behavioral aspects of animal welfare. Nevertheless, there is an immediate need to solve the challenges regarding the aerial environment in order to safeguard the intended welfare benefits of the recent ban on conventional cages. In order to introduce effective preventive measures against dust, there is a need for more knowledge of housing design, technical systems for ventilation and dust removal, litter material that produces little dust, and management routines. The large variation found within systems regarding levels of dust and its components indicate that improvements are within reach. For example, by spraying water with 10% rapeseed oil over the manure storage bins, dust concentration was reduced by 30%–45% [65]. Zheng *et al.* [76] showed that spraying slightly acidic electrolyzed water in an experimental aviary laying-hen housing chamber significantly reduced airborne culturable bacteria. On the other hand, the treatment did not succeed in reducing airborne particulate matter. Nevertheless, this is a promising technique for alleviating the adverse health impacts of bioaerosols in aviary laying-hen housing systems for both animal and workers. Ogink *et al.* [59] showed that spraying water in aviary air decreased the level of fine dust but enhanced ammonia emissions and odor. In conclusion, the use of aerosolized water (or solutions) should be investigated and refined as a possible method for the reduction of dust emission in laying hen systems. 

## 9. Conclusions

In two articles we have reviewed the available literature on potential welfare challenges related to high levels of dust (reported here) and ammonia (reported in Part II) in the alternative housing systems. There are gaps in knowledge on how laying hens react to dust, gases, and bioaerosols in the short and long term, what levels they find aversive and/or that impair health, as well as any additive or synergistic effects of dust and gases. The findings of Oyetunde *et al.* [73] show that there may be a substantial synergism in the effects of the various components that reduce the air quality. The uniqueness of the avian respiratory system means that studies conducted on mammals cannot readily be transferred to poultry. To find durable solutions to improve hen welfare in the new housing systems, the aerial environment has to be addressed. There is an urgent need for basic as well as applied research to reduce levels of dust and aerial pollutants in hen housing systems that are designed to increase welfare by allowing motivated behaviors in more complex environments. Thus, multi-criteria approaches that include information regarding hen health and behavior should be employed.
